# Visible Pulsed Laser-Assisted Selective Killing of Cancer Cells with PVP-Capped Plasmonic Gold Nanostars

**DOI:** 10.3390/mi14061173

**Published:** 2023-05-31

**Authors:** Aniket Mishra, Rafia Inaam, Shunya Okamoto, Takayuki Shibata, Tuhin Subhra Santra, Moeto Nagai

**Affiliations:** 1 Department of Mechanical Engineering, Toyohashi University of Technology, Toyohashi 441-8580, Japan; aniket.mishra.au@tut.ac.jp (A.M.);; 2 Institute for Research on Next-Generation Semiconductor and Sensing Science (IRES^2^), Toyohashi University of Technology, Toyohashi 441-8580, Japan; 3 Department of Engineering Design, Indian Institute of Technology Madras, Chennai 600036, India

**Keywords:** photothermal treatment, PVP capped gold nanostars, nanosecond pulse laser, less invasive

## Abstract

A new generation of nanoscale photosensitizer agents has improved photothermal capabilities, which has increased the impact of photothermal treatments (PTTs) in cancer therapy. Gold nanostars (GNS) are promising for more efficient and less invasive PTTs than gold nanoparticles. However, the combination of GNS and visible pulsed lasers remains unexplored. This article reports the use of a 532 nm nanosecond pulse laser and polyvinylpyrrolidone (PVP)-capped GNS to kill cancer cells with location-specific exposure. Biocompatible GNS were synthesized via a simple method and were characterized under FESEM, UV–visible spectroscopy, XRD analysis, and particle size analysis. GNS were incubated over a layer of cancer cells that were grown in a glass Petri dish. A nanosecond pulsed laser was irradiated on the cell layer, and cell death was verified via propidium iodide (PI) staining. We assessed the effectiveness of single-pulse spot irradiation and multiple-pulse laser scanning irradiation in inducing cell death. Since the site of cell killing can be accurately chosen with a nanosecond pulse laser, this technique will help minimize damage to the cells around the target cells.

## 1. Introduction

Cancer is one of the biggest causes of a high number of deaths worldwide. Advances in nanotechnology have opened the doors for new cancer therapy strategies in the previous two decades [1,2]. Gold nanoparticles have been utilized in a plethora of biomedical applications, such as bioimaging, biosensing [3], photothermal therapy, photodynamic therapy [4], antibacterial treatment [5,6], and drug delivery [7,8]. Gold nanoparticles offer excellent potential for biomedical applications due to their highly biocompatible nature and plasmonic properties. The plasmonic characteristics of nanoparticles are highly dependent upon the structure of a synthesized nanomaterial, and drastic changes in the plasmonic properties can be observed upon changing the surface morphology and synthesis method [9,10]. Numerous pointed branches of gold nanostars (GNS) create a “lightning rod” effect and considerably enhance the local electromagnetic field [11]. The considerable electromagnetic field increment causes strong light absorption properties and effective energy conversion for heating tumor cells in experiments using nanoparticle-mediated photothermal therapy. A combination of resonance enhancement and “the lightning rod effect” causes the large field amplification at the star’s tips due to the extreme curvature of nanostars where a stronger electric (E)-field and a higher charge density are produced. Therefore, nanostars can create E-field hotspots and are far more effective than relatively smoother particles, such as nanospheres, nanorods, and nanodiscs for applications such as surface-enhanced Raman scattering (SERS), bioimaging, and photothermal therapy (PTT) [12,13,14]. The plasmonics of GNS have been studied using a variety of analytical and empirical strategies, including label-free biosensing [15], two-photon photoluminescence, and SERS activity [16]. Different syntheses of gold nanostars involve the use of various non-biocompatible chemicals, such as cetyltrimethylammonium bromide (CTAB), which is cytotoxic and affects cell viability [17]. Hence a facile, reproducible, and biocompatible reactant-assisted synthesis of gold nanoparticles is needed for more optimum applications of gold nanoparticles in various biomedical applications, such as drug delivery, photodynamic therapy, and photothermal therapy.

PTT has special benefits in therapeutic strategies for cancer treatment, including high selectivity resulting in minimal invasiveness and precise spatial–temporal selection [18,19]. It can be compared to other strategies, such as immunotherapy, which have quite a low response rate and immune-related adverse effects, and chemotherapy, which can cause induce significant toxicity in healthy cells [20]. Initially, PTT was tested without photosensitizing agents; however, a high laser power was needed, and the requirement of integrated fiber cooling systems hampered the simple treatment system. A contrast agent was added to PPT, which allowed for the use of lower power lasers and system simplification of PPT. The laser wavelength is either in the visual spectrum using spherical gold nanoparticles or in the near-infrared (NIR) region using NIR-absorbing gold-based nanoparticles depending on the surface plasmon resonance (SPR) wavelength. CW NIR lasers are generally used for application in photothermal therapy because only a little supply of continuous laser light provides considerable heating. Huang et al. used various shapes of gold nanoparticles, including nanocages, nanospheres, and nanorods, for the photothermal therapy of cells by using NIR continuous wave (CW) lasers at different wavelengths [21]. Gold nanostars (GNS) were used for PTT with a NIR CW laser [22]. CW lasers have significant heat-affected zones and affect the viability of cells. The disadvantage of a heat-affected zone with a CW laser in photothermal experiments can be reduced by a short-pulse laser, such as femtosecond lasers [18,19,23], and nanosecond pulse lasers [24,25].

The combination of GNS and visible pulsed laser irradiation for PTT is promising, but it has not been reported yet. GNS were mostly irradiated in the IR range [12,14,26,27,28,29,30]. A visible nanosecond pulsed laser was used for GNP-assisted PTT [31,32]. Mahmoodazeh et al. used GNP@polymer-DOX theranostic medicine and demonstrated chemo-photothermal therapy of solid tumors [31]. Zharov et al. investigated laser-induced microbubbles around gold nanoparticles upon laser irradiation [32]. The heat conversion rate of gold nanoparticles in the visible region is better [13] than that of the NIR laser. The combination of GNS and visible pulsed laser irradiation is expected to establish an efficient and less invasive photothermal treatment.

This study aims to develop a low-cost, efficient, and less invasive cancer treatment that limits tumor development or eradicates tumors. We have prepared gold nanoparticles with star-shaped morphology via highly biocompatible one-pot synthesis. To increase the throughput of the process, we have used a visible nanosecond pulsed laser for the photothermal treatment of cancer cells. The nanoparticles were incubated over cancer cells, and upon irradiation with a laser, site-specific cell death was confirmed using fluorescence imaging.

## 2. Materials and Methods

### 2.1. Preparation of PVP-Capped Gold Nanostars

**Figure 1:**
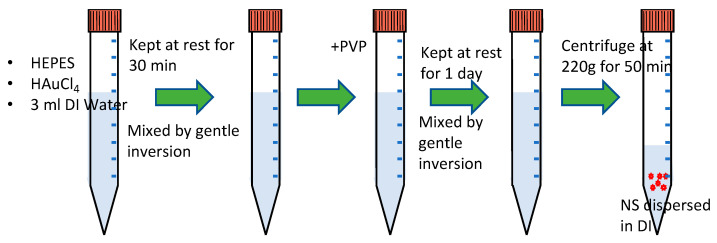
Schematic illustration of the synthesis of gold nanostars (GNS).

GNS were synthesized using a simple solution process (Figure 1) with some modifications [33]. A total of 3 mL of deionized water was mixed with 2 mL HEPES (4-(2-hydroxyethyl)-1-piperazineethanesulfonic acid, 100 mM). HEPES is Good’s Buffer and has minimal salt and temperature effects on the buffering capacity. HEPES produces nitrogen-centered free radicals and reduces gold ions, and it serves as an effective, weak reducing and capping agent [33]. HEPES concentration plays a crucial role in controlling the branched length of the particles and affects the shape and size of GNS [34]. A total of 20 µL of 50 mM HAuCl_4_ (chloroauric acid) was added, and the solution was mixed gently via inversion. The prepared solution was left to stand until a change in color from transparent to blue was observed, which took almost 30 min. The color change signifies a reduction of HAuCl_4_, and the change was monitored carefully, as it might affect the final concentration of GNS. Then, 600 µL of polyvinylpyrrolidone (PVP, 25 mM) was added and gently mixed. After 1 day, the solution was centrifuged at 220 g for 50 min to remove unadhered PVP from the GNS. The supernatant was removed, and the nanoparticles were suspended in DI water.

### 2.2. Cell Culture

HeLa cells were cultured in minimum essential medium (MEM, Gibco, 11095072, Thermo Fisher Scientific, Waltham, MA, USA) with 10% fetal bovine serum (FBS, CCP-FBS-BR-500, Cosmo Bio, Tokyo, Japan) and 1% penicillin-streptomycin (10,000 U/mL, Thermo Fisher Scientific, Waltham, MA, USA) for 3 days. The adherence of HEK-293 cells to the Petri dish was relatively weak; hence, the glass Petri dish was coated with fibronectin before seeding cells. For HEK-293 cells, glass Petri dishes were first coated with fibronectin and placed in humidified atmosphere incubator with 5% CO_2_ at 37 °C. After coating the dish with fibronectin, HEK-293 cells were cultured. Both HEK-293 and SAOS-2 cells were cultured using high glucose DMEM (FUJIFILM Wako Pure Chemical Co., Ltd., Tokyo, Japan) with 10% FBS and 1% penicillin–streptomycin (PS).

### 2.3. Procedure for the Site-Specific Killing of Cancer Cells

The medium of cultured cells was removed, and cells were washed using phosphate buffer saline (PBS) twice. Then, a new colorless modified Dulbecco’s modified eagle medium (DMEM) was used for cancer cell killing experiments. After the fresh medium was added, the cells were incubated with 4 different concentrations of PVP-capped GNS, *ρ* = 20 µg/mL, 15 µg/mL, 10 µg/mL, and 5 µg/mL, named, respectively, *ρ_A_* to *ρ_D_*, and incubated in 5% CO_2_ supply incubator for 4 h. Then, the medium was removed, and cells were washed with PBS twice to remove unadhered PVP-capped GNS. After washing, fresh DMEM was added. Then, 4 µM of propidium iodide (PI) was added for indication of laser-induced cancer cell killing. To observe cell viability, the medium was removed, and cells were washed with PBS twice. A new medium was added followed by the addition of Calcein-AM. The cells were observed with an inverted microscope (Nikon, ECLIPSE Ti-E, Tokyo, Japan) and microscope camera (Nikon, DS-Fi3).

### 2.4. Experimental Setup

**Figure 2:**
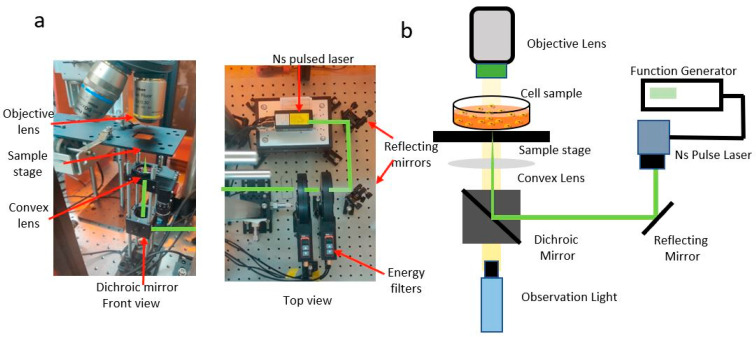
Experimental setup for site-specific selective killing of cancer cells. (**a**) Photo and (**b**) schematic of the system.

The experimental setup consisted of irradiation, scanning, and observation systems (Figure 2a,b).

Irradiation system: We used a nanosecond pulsed laser (Cobolt, Tor^TM^ XS, Hubner Photonics, Solna, Sweden) with a Gaussian profile, a wavelength of 532 nm, a pulse of 5 ns, and a frequency of 1 kHz. The laser was controlled with a digital function generator. The laser light was reflected through a mirror for alignment. A dichroic mirror (Thorlabs, DMLP550R, Newton, NJ, USA) reflected the laser and combined observation light. The original diameter of the laser beam was 1.1 ± 0.3 mm, and a convex length of focal length of 50 mm focused the laser on a sample.

Scanning system: A cartesian robot (IAI Corporation) was used for scanning a sample. The scanning speed was kept constant at 100 mm/s for all the experiments.

Observation system: The samples were observed using a CMOS (ZWO, ASL1600MC-Cool, Suzhou, China) camera and an observation light (Thorlabs, MWWHLP1). The samples were visualized using the ImageJ webcam plugin.

### 2.5. Data Analysis

The cell killing efficiency of spot irradiation was calculated from an average of five irradiation spots for each experimental condition. All the experiments were performed in triplets, and efficiency was reported as mean ± standard deviation for three independent experiments. In the case of scanning laser, the efficiency was calculated by counting the number of cells along the laser path for a distance of 1000 µm for 3 different scan paths for each parameter for a single experiment, and the reported efficiency is the mean ± standard deviation of 3 independent experiments. No effect of laser on cells leads to 0% cell killing, and peeling off of cells upon laser irradiation is defined as 100% cell killing efficiency. To evaluate cell killing efficiency in the case of single pulse spot irradiation, the number of dead cells and live cells within the laser circumference of the irradiated spot were calculated, and the efficiency was evaluated. For continuous pulse laser-induced cancer cell killing, the cell killing efficiency was evaluated by counting the number of dead cells and live cells along the path of laser irradiation, and the efficiency η was evaluated as follows (except peeling off of cells at higher laser energies):

$$\eta =\frac{N_{R}}{N_{R}+N_{G}}\times 100$$

where *N_R_* and *N_G_* are, respectively, the numbers of PI-stained and calcein-AM-stained cells.

## 3. Simulation of Temperature Rise in Gold Nanostars upon Laser Irradiation

### 3.1. Simulation Method

We performed heat transfer simulations in COMSOL Multiphysics to confirm that the heat upon laser irradiation on GNS is generated more than on gold nanospheres. Such higher heat generation allows for site-specific killing at a lower laser energy, resulting in less effect on cells in the vicinity. A 2D Gaussian laser pulse irradiated on gold nanoparticles is modeled by [35].

$$S(t)=\frac{0.94\times F_{abs}}{\delta t_{p}}\exp\left[-4\ln(2){\left(\frac{t-2t_{p}}{t_{p}}\right)}^{2}\right]$$

$Q_{in}$ represents total heat load, $Q_{o}$ represents total input power, $R_{c}$ is the reflection coefficient, $A_{c}$ is the absorption coefficient within *x* and *y* coordinates, $\sigma_{x}$ is the standard deviation in the x-direction, and $\sigma_{y}$ is the standard deviation in the y-direction. To obtain the temperature rise of gold in both shapes, two temperature models were considered [2,28]. An irradiated laser pulse resulted in a rise in the temperature of electron layer *T_e_* in the respective specimen. The heat from the electron layer was transferred to a gold lattice covered with polyvinyl pyrrolidone, which rose the lattice temperature *T_l_*. Finally, the heat was dissipated to the water body into which the nanoparticles were immersed separately. A temperature rise *T_m_* of the water bodies was observed.

$$C_{e}\left(T_{e}\right)\frac{{\partial T}_{e}}{\partial t}=\nabla \left(k_{e}\nabla T_{e}\right)-g\left(T_{e}-T_{l}\right)+Q(t)$$

$$C_{l}\frac{{\partial T}_{l}}{\partial t}=\nabla \left(k_{l}\nabla T_{l}\right)-g\left(T_{l}-T_{e}\right)+Q(t)$$

$$\rho_{m}\left(r\right)c_{m}\left(r\right)\frac{\partial T_{m}\left(r,t\right)}{\partial t}=\nabla .\left(k_{m}.\nabla T_{m}\left(r,t\right)\right)+F(t)$$

where $C_{e}\left(T_{e}\right)=\gamma T_{e}$ with $\gamma =70\;Jm^{-3}K^{-2}$ is the electron heat capacity of gold, $C_{l}$ is the lattice heat capacity of gold, $k_{e}=300\;Wm^{-1}K^{-1}$ [8] is electron thermal conductivity, $k_{l}=2.7\;Wm^{-1}K^{-1}$ [1] is the lattice thermal conductivity, $g=2\times 10^{16}\;Wm^{-3}K^{-1}$ [8] is the gold electron phonon coupling element, $C_{m}=4182\;Jm^{-3}K^{-1}$ [8] is the water heat capacity, and $k_{m}=0.6\;Wm^{-1}K^{-1}$ is the water thermal conductivity. The laser was irradiated at a fluence of 100 mJ/cm^2^. The function *F(t)* represents the heat transfer at the interface between the GNS and surrounding area. All parameters were obtained from [8].

### 3.2. Simulation Results and Discussion

**Figure 3:**
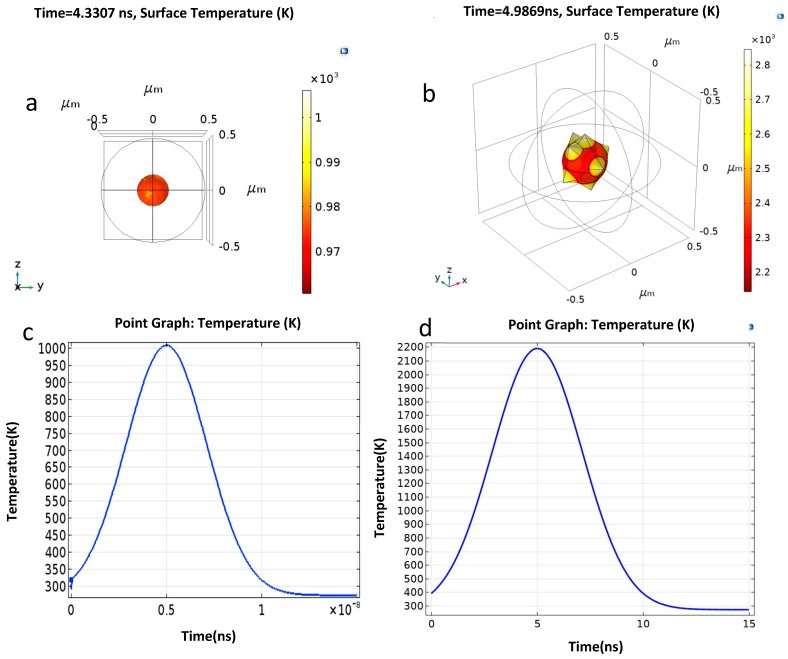
(**a**)Temperature rise in gold nanosphere. (**b**) Temperature rise in star-shaped gold nanoparticle. (**c**) Maximum temperature rise of nanosphere core. (**d**) Maximum temperature rise of the core of a star-shaped nanoparticle.

The simulation results in Figure 3a,b show the temperature rise in the star-shaped nanoparticles. The figures show that the temperature rise in a star shape is almost double the temperature rise in a sphere shape. In Figure 3b, an interesting phenomenon of a higher temperature in star spikes as compared to a star core can be seen. This is because spikes primarily come in contact with laser heat during heating, whereas certain areas of the cone in contact with the base of the spikes do not directly receive heat. Moreover, it can be seen that the surface area of a gold nanostar is larger compared to a gold nanosphere. Due to the higher surface area, heat gain is also increased. Due to these reasons, there is localized heating in spikes on GNS, which overall increases the temperature of a particle. This explains the reasons for almost double the temperatures observed on GNS compared to nanospheres.

Figure 3c,d indicates the temperature rise in sphere- and star-shaped nanoparticles against time. The simulation time for the gold nanosphere and nanostar was 15 ns. The maximum temperature for a gold nanostar was 2200 K, and for a nanosphere, it was 1000 K. By comparing all the results, we can see that the temperature rises and falls for a star shape are much larger than for a sphere shape; this may be because there is heat accumulation in the star spikes, leading to localized heating and resulting in higher temperature rises.

## 4. Experimental Results

### 4.1. FESEM Analysis and UV–Visible Spectroscopy Analysis

**Figure 4:**
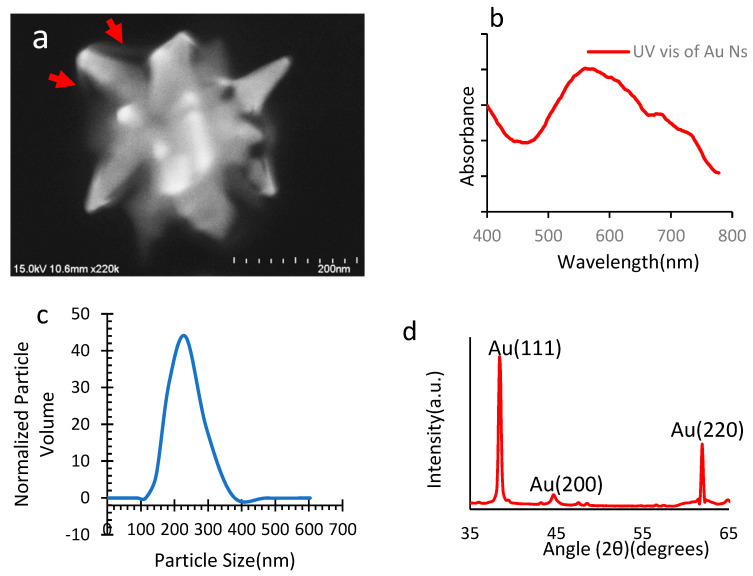
Characterization of PVP-capped GNS. (**a**) FESEM image. (**b**) UV–visible spectrum. (**c**) Particle size distribution. (**d**) XRD of particles on a silicon wafer.

The prepared nanomaterials were characterized using field emission scanning electron microscopy (FESEM) for morphological analysis. Figure 4a represents PVP-capped gold nanoparticles in star morphology where arrows indicate the capping; the pointed tips appearing in the image provide hotspots for field enhancement on plasmonic gold nanoparticles. Hotspots are the specific locations on NPs where electric field enhancement occurs upon laser illumination. GNS offer more hotspots in comparison to other shapes, such as nanotubes or nanospheres. A PVP cap can be visually observed in FESEM. The arrows indicate PVP caps. Our nanosecond pulsed laser has a wavelength of 532 nm, and we checked that the prepared nanoparticles absorbed the laser light around this wavelength under UV–vis spectroscopy. UV–vis (Figure 4b) shows that the prepared nanoparticles absorbed the given laser light with a maximum absorption peak of around 532 nm.

Smaller nanoparticles have been reported to get transported into the cell cytosol via endocytosis [36]; hence, we have preferred to use particles with relatively bigger sizes that allow for cell killing to occur due to ion imbalance because of irreversible pore creation on the cell membrane. Commonly nanoparticles with a size of 200 nm have been used for photothermal biomedical experiments [37,38]. The size of gold nanostars as measured using a laser diffraction particle size analyzer (SALD 2300, Shimadzu, Kyoto, Japan) revealed that 90% of particles were under 262 nm, whereas the mean particle size was 204 nm, as depicted in Figure 4c.

The X-ray diffractometer (XRD, Rigaku ULTIMA IV R185, Tokyo, Japan) analysis (Figure 4d) of the nanoparticles on a silicon wafer revealed the presence of peaks at 38.46°(1 1 1), 44.78°(2 0 0), and 62.1°(2 2 0), which is identical to standard gold metal (Au^0^) with respect to JCPSD no. 04-0784. XRD analysis indicates the presence of face-centered cubic (FCC) structure gold (Au). The shift of gold peaks was almost negligible compared to the standard data. The absence of significant additional peaks of impurities indicates that no crystal impurities were present in the form of crystal defects in the face-centered cubic structure of gold.

### 4.2. Cancer Cell Killing Analysis

The cancer cell killing efficiency of the visible pulse laser-assisted setup was evaluated by two methods: single pulse irradiation and scanning multiple pulse irradiation. The biocompatibility of PVP-capped GNS was analyzed by using an MTT assay (Supplementary Section S3 and Figure S7).

#### 4.2.1. Site-Specific Cancer Cell Killing with Single Pulse

**Figure 5:**
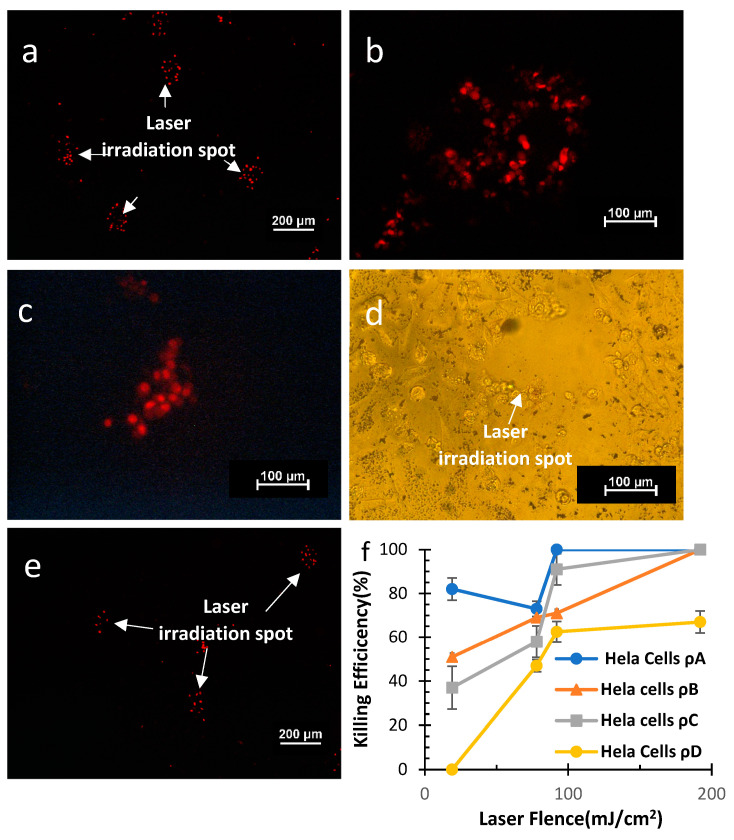

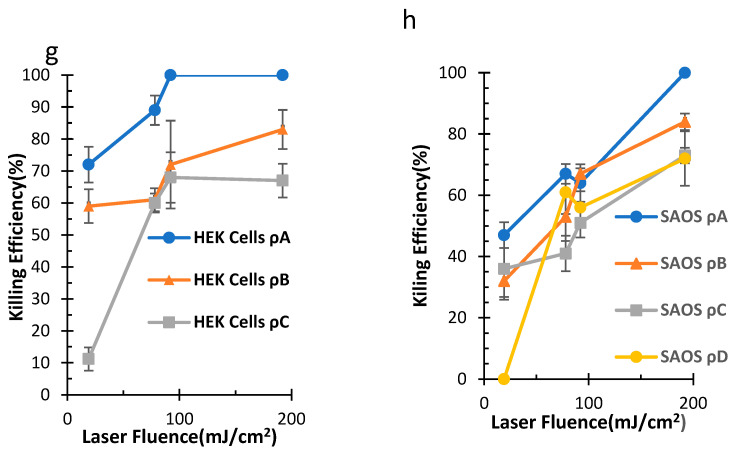
Single pulse spot irradiation killing. HeLa cells (**a**) at 192 mJ/cm^2^ at *ρ_D_* and (**b**) 192 mJ/cm^2^ at *ρ_D_.* (**c**) HEK-293 cells at 92 mJ/cm^2^ at *ρ_C_*. (**d**) Peeling off of HEK-293 cells at 92 mJ/cm^2^ at *ρ_A_*. (**e**) Spot irradiation of SAOS-2 cells at 192 mJ/cm^2^ at *ρ_B_*. Cell killing efficiency vs. laser fluence curve at various concentrations for (**f**) HeLa cells, (**g**) HEK-293 cells, and (**h**) SAOS-2 Cells.

Fluorescence microscopy was used to examine cancer cells that had been killed at specific sites. HeLa cells at the highest concentration (*ρ_A:_* 20 µg/mL) in case of spot irradiation were peeled off from the surface of the glass bottom Petri dish due to the high magnitude of shock wave generation upon laser irradiation (Figure 5d), whereas the cells at the other concentrations (*ρ_B_:* 15 µg/mL, *ρ_C_*: 10 µg/mL) unadhered only at the highest laser fluence of 192 mJ/cm^2^. The efficiency of cell death was assessed at lower fluences (Figure 5a–c), and as laser fluence and particle concentration increased, so did the efficiency of cell death (Figure 5f). The cells were unaffected by the laser irradiation at the lowest concentration (*ρ_D_:* 5 µg/mL) and the lowest laser fluence of 19 mJ/cm^2^ due to an insufficient amount of laser effect.

Next, we tested the efficacy of our system for site-induced cell killing in the HEK-293 cell line. Single pulse laser irradiation at higher energies at *ρ_A_* detached the HEK-293 cells from the substrate similar to that of HeLa cells. At lower concentrations (*ρ_B,_ ρ_C_*), the cell death efficiency increased upon increasing the laser fluence. Upon decreasing the particle concentration from *ρ_B_* to *ρ_C_* (Figure 5g), the cell killing efficiencies were comparable at 78 mJ/cm^2^ and 92 mJ/cm^2^ (Figure 5c) and different at maximum and minimum laser fluence. For the lowest concentration of photosensitizer nanoparticles (*ρ_D_*), the cells were unaffected by laser irradiation. This implies that a laser with various fluences at concentration is unable to generate enough vapor nanobubbles essential for the generation of the shock wave to induce pores on the cell wall where the pores possibly create an electrolyte imbalance and induce cell death due to heterogeneity found in different cell lines (HeLa and HEK-293).

We tested our system on a human osteosarcoma cell line (SAOS-2) and verified that our method can be applied to various cancer cell lines (Figure 5h). Increasing both the laser fluence and particle concentration increased the cell killing efficiency. As a general trend, a notable change in efficiency was observed at changing the laser fluence from 19 mJ/cm^2^ to 78 mJ/cm^2^, whereas the efficiency was almost similar upon further increasing the laser fluence from 78 mJ/cm^2^ to 92 mJ/cm^2^. As expected, the highest cell killing efficiency was observed at 192 mJ/cm^2^ (Figure 5e). The cell killing efficiencies at *ρ_B_* and *ρ_C_* were almost comparable with respect to various fluences. The cells at *ρ_A_* were observed to detach at a fluence of 192 mJ/cm^2^. In general, it can be concluded that the cancer cell killing efficiency increases significantly upon increasing the laser fluence and the photosensitizer nanoparticle concentration.

#### 4.2.2. Site-Specific and High throughput Scanning Laser-Induced Cell Killing

**Figure 6:**
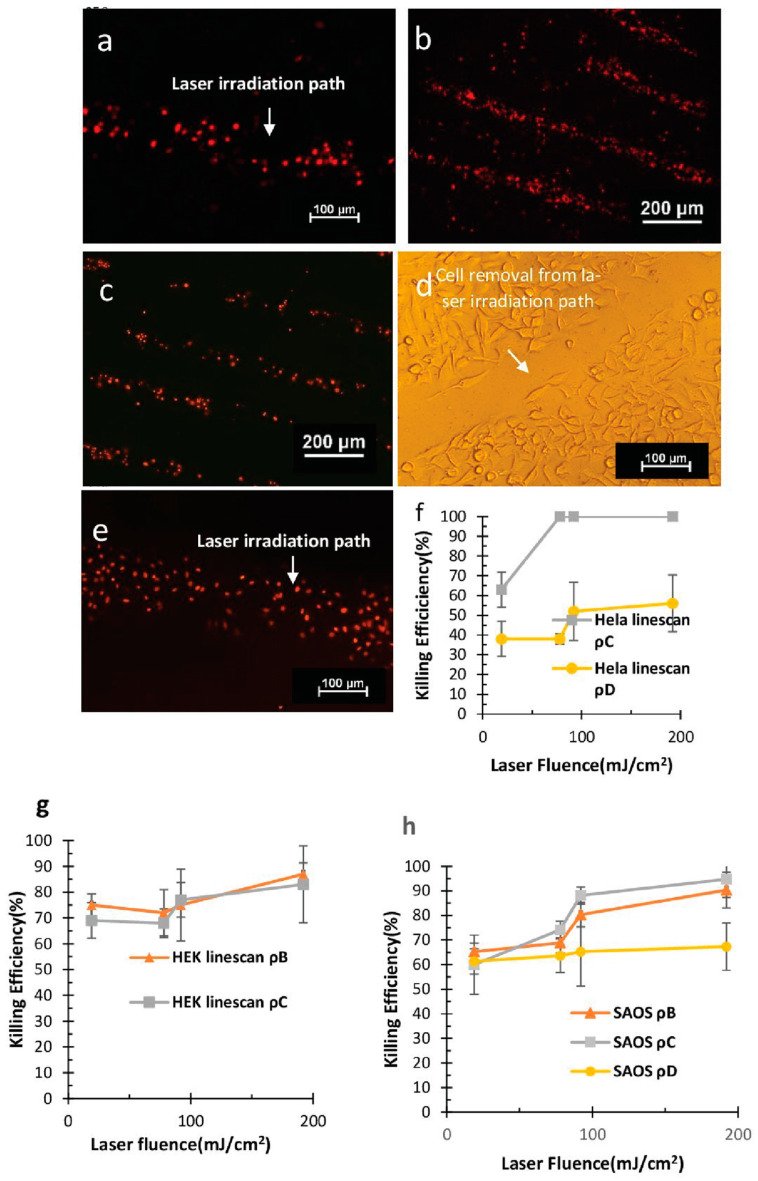
High Throughput scanning laser irradiation killing in HeLa cells (**a**) at *ρ_C_* at 92 mJ/cm^2^ (**b**) at *ρ_B_* at 78 mJ/cm^2^ (**c**) at *ρ_C_* at 78 mJ/cm^2^. (**d**) Peeling off of cells at higher Laser fluence at *ρ_A_* at 78 mJ/cm^2^. (**e**) Scanning laser irradiation SAOS-2 cells at 192 mJ/cm^2^ at *ρ_B_*. Cell killing Efficiency vs. Laser Fluence curve at various concentrations for (**f**) HeLa cells, (**g**) HEK-293 cells, and (**h**) SAOS-2 cells.

Continuous pulsed laser irradiation led to the irradiation of multiple laser pulses at a single spot, and the magnitude of the generated shock wave was stronger. At the highest concentration (*ρ_A_*), cells were peeled off at all the laser fluences (Figure 6d and Supplementary Figures S1 and S2). While at lower nanoparticle concentrations (*ρ_C_, ρ_D_*), high throughput site-specific HeLa cell killing was observed (Figure 6a,b), and the results were similar to spot irradiation, indicating an increment in cell killing efficiency when either the laser fluence or the nanoparticle concentration was increased. At *ρ_C_*, 100% cell killing efficiency was observed at higher laser fluences. There was a significant difference in cell killing efficiency at *ρ_C_* and *ρ_D_* (Figure 6f). In the case of HEK cells, the continuous pulse laser irradiation of cells on the glass substrate resulted in the peeling of cells at the highest photosensitizer nanoparticle concentration (*ρ_A_*), thus resulting in 100% cell killing efficiency at all the laser fluences. For the other concentrations (*ρ_B_, ρ_C_*) (Figure 6c), the cells were killed upon laser irradiation, and the efficiency was calculated. The cell killing efficiency was observed to be gradually increasing upon increasing the laser fluence, while the effect of decreasing the nanoparticle concentration (*ρ_B_, ρ_C_*) on the killing efficiency was negligible, as depicted in Figure 6g.

SAOS-2 cells were peeled off at the highest concentration of photosensitizer nanoparticles (Figure S3). Figure 6e represents the high throughput killing of SAOS-2 cells at 192 mJ/cm^2^ at *ρ_B_*. At *ρ_B_* and *ρ_C_*, the cell killing efficiency was found to increase in a similar fashion to spot irradiation for the same cell line. That is, a gradual increase in the cell killing efficiency was observed upon increasing the laser for both concentrations (Figure 6h), while for *ρ_D_*, the cell killing efficiency was almost comparable at various laser fluences. We can conclude that upon increasing particle concentration and laser energy, an increase in cell killing efficiency has been observed; the reason for which will be further elaborated in the discussion section.

In this study, the Meander pattern was used to differentiate between dead cells and live cells. On the other, reducing the step size of laser scanning a large number of cells in a bigger region of interest can also be processed for high throughput specifically (Figure S4). Apart from these regions, scanning laser irradiation at the parameters in the middle range of no cell death and cell death. Delivery of PI dye was observed into cell cytoplasm in the case of both HeLa and HEK-293 cell lines (Figure S5).

#### 4.2.3. Control Experiments

Control experiments were carried out to confirm PVP-capped GNS-assisted visible pulsed laser-induced site-specific killing of cancer cells. The laser was not irradiated to a sample and cells incubated with PVP-capped GNS control images were taken (Figure S6a,b Hela cells). Cells without photo absorber incubation and laser irradiation(Figure S6c,d HEK cells). Most cells were alive in both cases, which concludes that both particle incubation and laser irradiation are necessary for killing cancer cells.

## 5. Discussion

The potential use of nanomaterials in a PTT strategy against cancer cells has been noticed in several studies. Most of these studies have concentrated on using NIR to locally increase temperature and selectively induce cell death. However, visible light irradiation for PTT has been disregarded despite the greater visible light-to-heat conversion that occurs when nanoparticles are irradiated with a laser, and only a few studies have reported the use of visible pulsed lasers in biomedical applications [39,40]

Several parameters play a crucial role in the photothermal treatment of cancer with respect to photosensitizer particles; for example, the absorption efficiency for nanoparticles with a branched structure is more than that of spherical particles [41]. The size of nanoparticles can also affect the absorption efficiency, as smaller particles tend to absorb more light. The danger of adjacent cell damage and endocytosis is present; hence, for biomedical photothermal experiments, relatively bigger particles are preferred [39,42]. Similarly, concentration also plays a crucial role in photothermal efficiency, while lower concentration results in insufficient absorption of light for treatment, while high concentration can result in damage to adjacent non-target cells.

The laser energy absorption of GNS can be explained by SPR. The prepared GNS absorbed the laser and converted it into heat. Efficient conversion needs the overlap of the absorption spectrum and the laser wavelength; less overlap reduces the absorbance. The laser wavelength 532 nm was in the absorbance region of the prepared GNS with a peak of 550 nm. Our study used a visible pulsed laser for the PTT of cancer cells. In addition to a nanosecond pulse laser, picosecond and femtosecond pulse lasers can be irradiated to GNS and are considered to lead to a smaller heat-affected zone.

**Figure 7:**
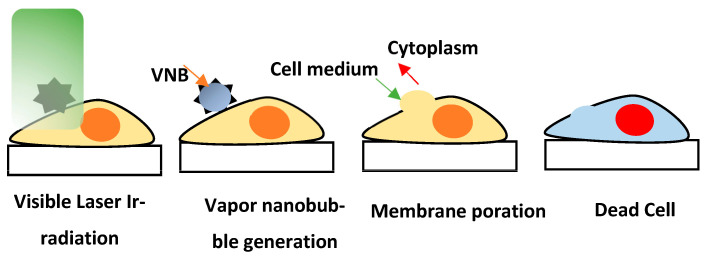
Schematic of cell death mechanisms.

According to the above model mentioned in the simulation results, when PVP-capped GNS are exposed to a nanosecond pulsed laser beam, the laser exposure raises the temperature of the electrons inside the GNS. This temperature rise in turn increases the lattice temperature of the GNS. After only a few picoseconds, the medium’s temperature starts to rise because of electron–phonon interaction [43]. This increase in temperature causes the surrounding medium to produce vapor nanobubbles [44,45,46], which, when collapsed, cause stress on the membrane and generate transitory pores. Cell death results from an imbalance in the concentration of electrolytes after the formation of holes in the cell membrane [47,48], as depicted in Figure 7. The other possible mechanism for cell death is heat transfer between GNS [49,50,51] and the cell membrane. Upon heat transfer to the cell membrane, local phase transition may occur in the lipid bilayer. Thermal denaturation of glycoproteins can take place [52], which might result in the opening of transient hydrophilic pores on the cell membrane, thus creating the electrolyte imbalance and initiating cell death.

The usual tumor size ranges from millimeters to centimeters, and for the site-specific killing of cancer cells, a highly fluent laser beam was focused on the sample by using a convex lens in dimensions of micrometers [53]. Certain skin pre-malignancies and cancers, as well as pre-cancers or extremely early stages of the cervix and nearby cancers, can be treated using this strategy of cancer therapeutics. Inside the body, cancers obstructing the airway and progressing from other locations to the lungs can be treated with lasers. Tumors in the breast, brain, skin, head, neck, cervical area, and lung can be eliminated using precise lasers; thus, this PTT can be used for the treatment of the same organs.

## 6. Conclusions

In this paper, a nanosecond pulse laser was used to increase the throughput of the process, as it can be utilized for selectively killing cancer cells in a small duration of time to precisely eliminate cancer cells with PVP-capped GNS. The nanostars were made using facile synthesis methods, and their characteristics were determined using FESEM, UV–visible spectroscopy, and XRD analysis. Cancer cells cultivated on a glass Petri dish were incubated with nanoparticles. The cells were exposed to the nanosecond pulse laser, and cell death was confirmed by observing PI-stained cell nuclei under a fluorescence microscope. The effect of the variation of cell killing efficiency with respect to laser fluence and particle concentration was analyzed, and we successfully analyzed three cell lines (HeLa, HEK-293, and SAOS-2). The efficiency for single spot and high-throughput laser scanning cell killing was evaluated.

## Data Availability

Not applicable.

## Supplementary Materials

The following supporting information can be downloaded at: https://www.mdpi.com/article/10.3390/mi14061173/s1, Figure S1: Peeling off of cells at 192 mJ/cm^2^ at *ρ_A_*; Figure S2: Peeling off of HEK-293cells at 78 mJ/cm^2^ at Conc. A; Figure S3: Peeling off of SAOS-2 cells at 78 mJ/cm^2^ at Conc. A; Figure S4: Parallel processing of larger region of interest using reduced step size for laser scan; Figure S5: (a) PI-delivered HeLa cells, (b) viable HeLa cells, (c) PI-delivered HEK-293 cells, and (d) viable HEK-293 cells. Figure S6: Control image without laser irradiation. (a) Dead HeLa cells (PI staining), (b) live HeLa cells (Calcein-AM staining), and without photo absorber, (c) dead HEK-293 cells, and (d) live HEK-293 cells. Figure S7: MTT assay analysis of gold nanostars for 4 h, 24 h, and 48 h.
