# Supplementary material for: Visible Pulsed Laser-Assisted Selective Killing of Cancer Cells with PVP-Capped Plasmonic Gold Nanostars
Source: Micromachines (Basel). 2023 May 31;14(6):1173. doi: 10.3390/mi14061173 (PMC10305603; doi:10.3390/mi14061173)
Supplement: Supplementary file 1 [file micromachines-14-01173-s001.zip › micromachines-2417019-supplementary.pdf]

Supplementary material

Visible Pulsed Laser-assisted Selective Killing of Cancer Cells with PVP-capped Plasmonic Gold Nanostars

Aniket Mishra [1], Rafia Inaam[1], Shunya Okamoto[1], Takayuki Shibata [1,2,] Tuhin Subhra Santra,[3] and Moeto Nagai [1,2*]

[1]Department of mechanical Engineering, Toyohashi University of Technology

[2]Institute for Research on Next Generation Semiconductor and Sensing Science(IRES[2]), Toyohashi University of Technology

[3] Department of Engineering Design, Indian Institute of Technology, Madras

* Correspondence: nagai@me.tut.ac.jp

1. Peeling off of cells after laser irradiation

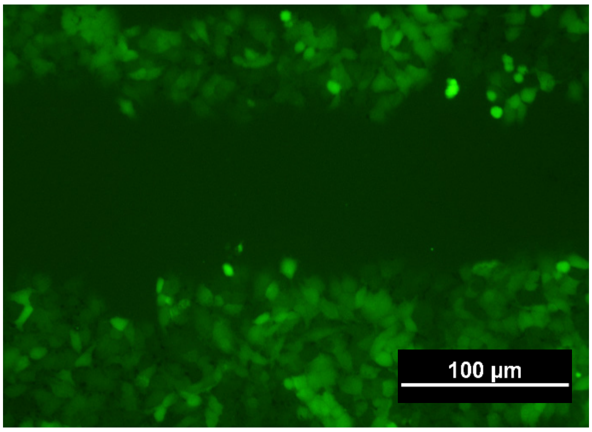

Figure S1 Peeling off of HeLa cells at 192mJ/cm^2 at Conc. A

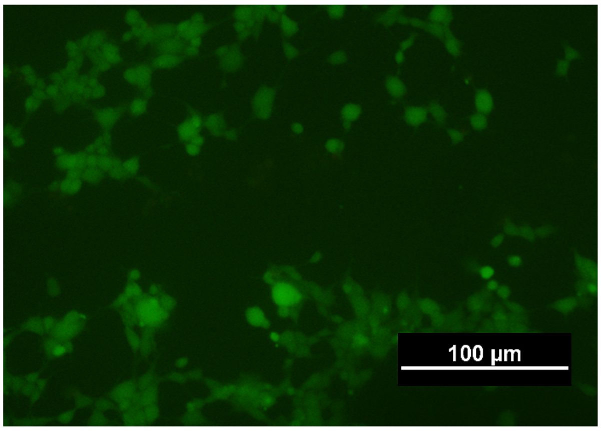

Figure S2 Peeling off of HEK-293cells at 78mJ/cm^2 at Conc. A

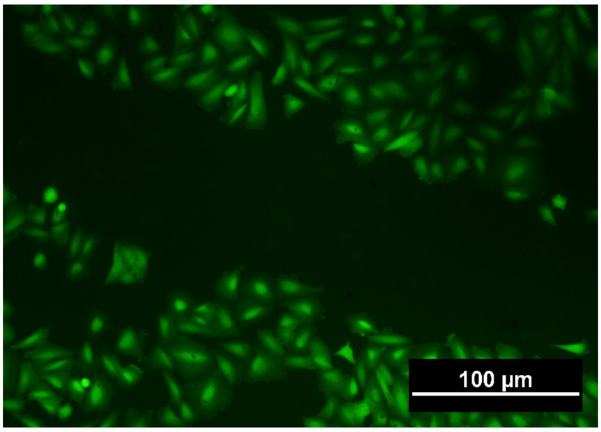

Figure S3 Peeling off of SAOS-2 cells at 78mJ/cm^2 at Conc. A

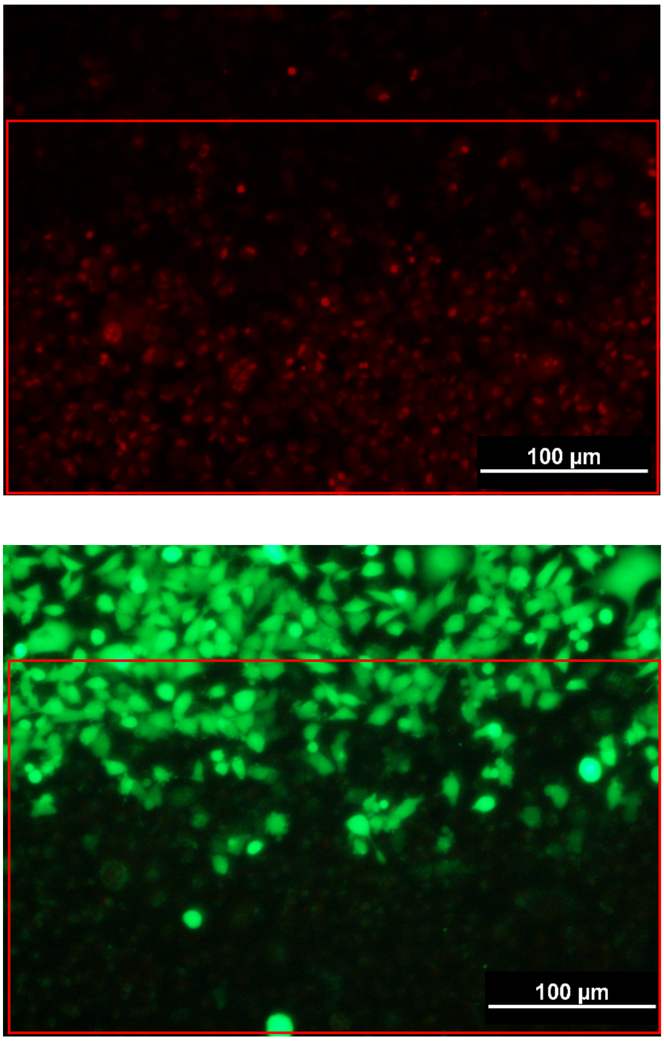

Figure S4 Parallel processing of larger region of interest using reduced step size for laser scan (Rectangle represents the scanned area).

2. Dye Delivery into cells:

Interestingly, at certain parameters instead of killing HeLa cells, the cytoplasm of Hela cells was stained with PI dye Normally, when ns laser falls over photoabsorber incubated cells, pores are created on the cell membrane, and electrolyte imbalance occurs thus resulting in cells death. However, these membrane pores are resealable in nature so when the pores are able to seal before electrolyte imbalance occurrence, the result is the delivery of cell membrane impermeable PI dye into the cell cytoplasm. Interestingly at a laser fluence of 50mJ/cm2 instead of killing cancer cells, The laser delivered PI into the cancer cells at concentration D for HeLa cells (Figure S5a and S5b) while for HEK cells Dye was delivered at 50mJ/cm2, 78mJ/cm2 (Figure S5c and Figure S5d and 192 mJ/cm^2. Note that the stepsize of scanning was reduced to scan the whole sample.

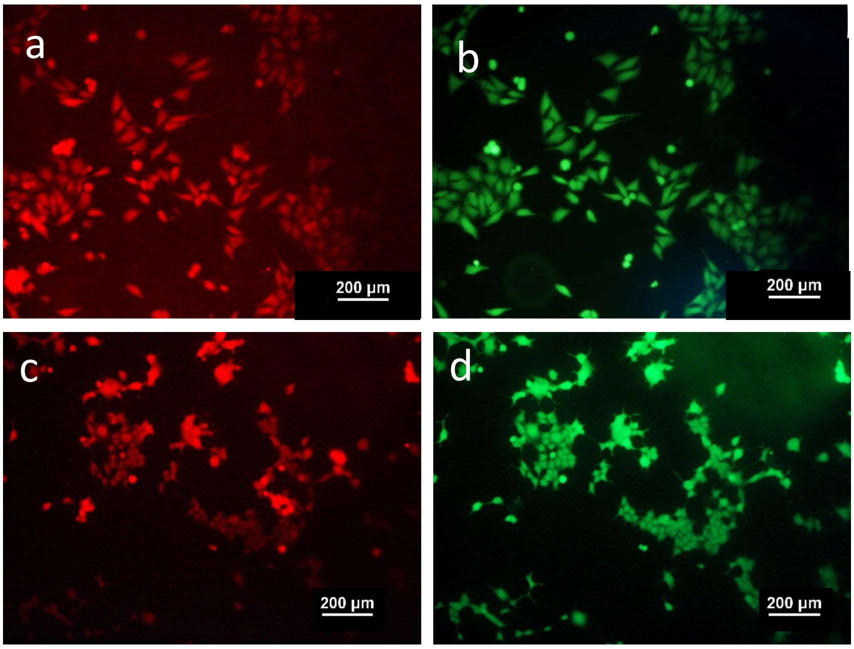

Figure S5 (a)PI delivered Hela cells. (b) Viable Hela cells. (c) PI delivered HEK-293 cells. (d) Viable HEK-293 cells.

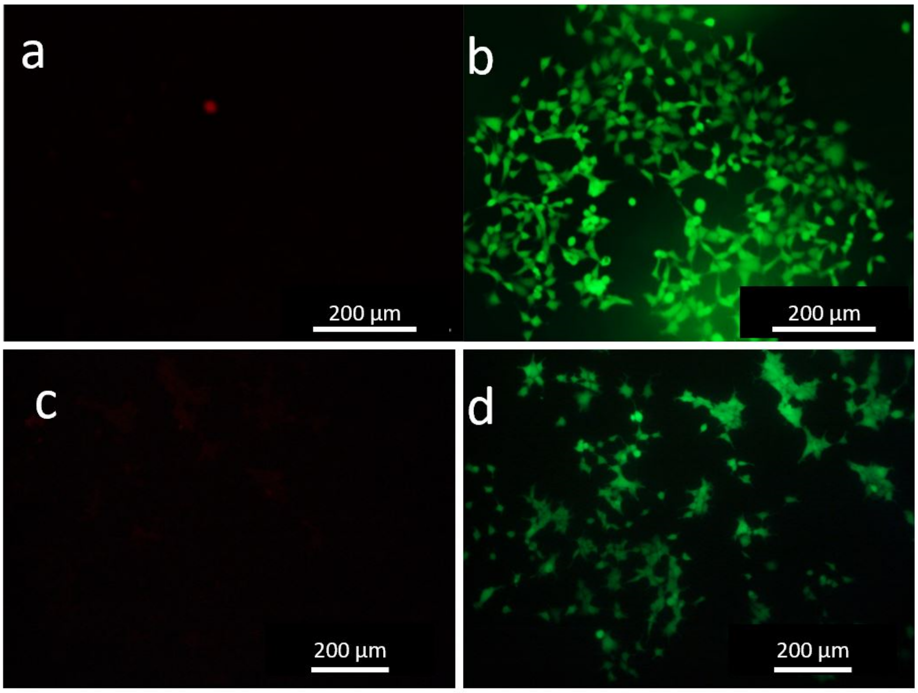

Figure S6. Control image without laser irradiation with photo absorber. (a)Dead cells (PI staining) and (b) live cells (Calcein AM staining) Hela cells and with laser irradiation and no photo absorber. (c) Dead cells (d) live cells (HEK-293 cells).

3. Biocompatibility analysis

The biocompatibility analysis of the prepared nanoparticles was done by MTT assay (3-[4,5-dimethylthiazol-2-yl]-2,5 diphenyl tetrazolium bromide, Analytical Grade, TCI, Japan) on HeLa cells. 2×10^4 Hela cells were seeded in the 96 well plate for culturing overnight in a humidified incubator with 5% CO_2 supply at 37°C. The medium was removed, and cells were washed with PBS followed by the addition of a new medium and nanoparticles at various concentrations. After 4 hours of incubation, the cells were washed thrice with PBS and followed by the addition of new MEM and 10 µl MTT at 5mg/ml concentration. After four hours of incubation, the medium containing MTT was removed and 200µl dimethyl sulfoxide (Sigma Aldrich) was added in every well to dissolve formazan crystals. The MTT assay was evaluated after 4 hours, 1 day and 2 days after nanoparticle incubation over cells. The optical density was recorded at 570 nm through an "enzyme-linked immunosorbent assay (ELISA)" plate reader (AS ONE, MPR-A 100). The cell viability is very high at all the used concentrations as evidenced by Figure S7 which indicates the viability of cells relative to control cells without nanoparticle incubation is almost equal to 100% indicating the biocompatible nature of the prepared nanoparticles.

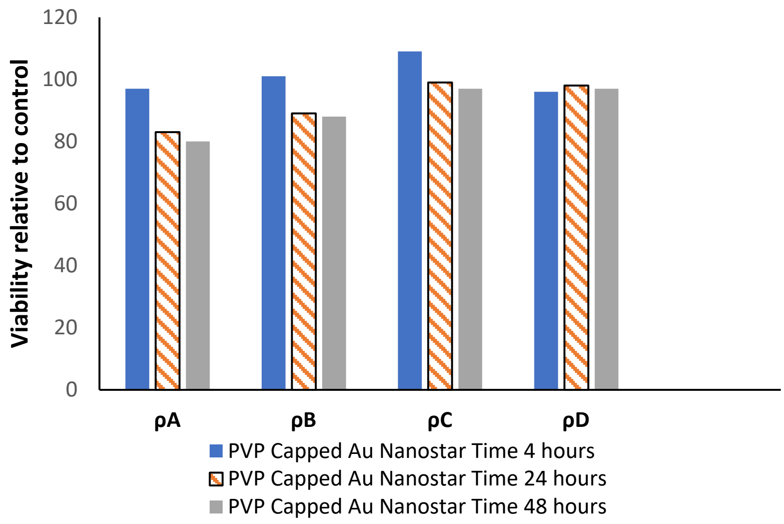

Figure S7 MTT assay analysis of Gold nanostars for 4 hours, 24 hours, and 48 hours
